# Supplementary material for: Unveiling a Shift in the Rotavirus Strains in Benin: Emergence of Reassortment Intergenogroup and Equine-like G3P[8] Strains in the Post-Vaccination Era
Source: Viruses. 2025 Aug 7;17(8):1091. doi: 10.3390/v17081091 (PMC12390709; doi:10.3390/v17081091)
Supplement: Supplementary file 1 [file viruses-17-01091-s001.zip › viruses-3705211-supplementary figures.pdf]

viruses

MDPI

Supplementary Material

Unveiling a shift in the rotavirus strains in Benin: Emergence of reassortment intergenogroup and equine-like G3P[8] strains in the post-vaccination era

3. Results

3.2. Phylogenetic analysis

3.2.3. Phylogenetic analysis of VP1-3 and VP6

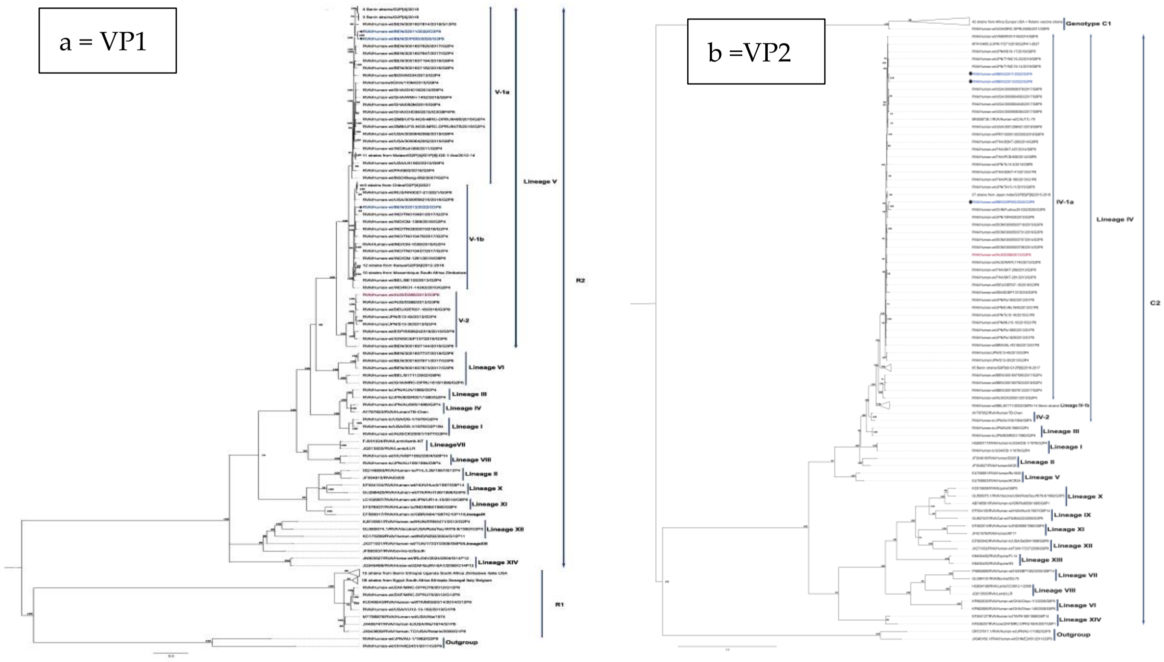

c =VP3

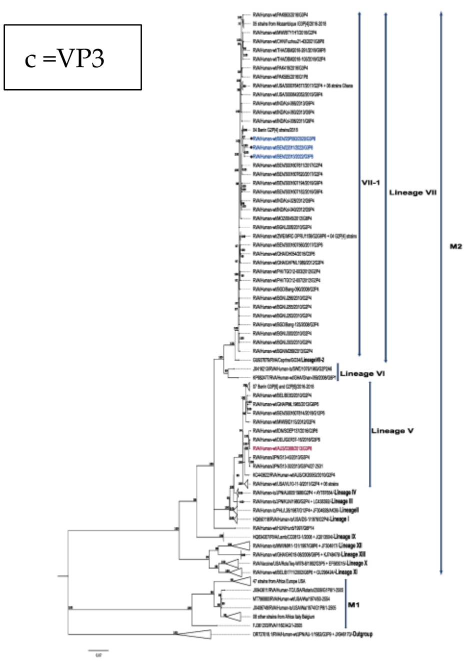

d =VP6

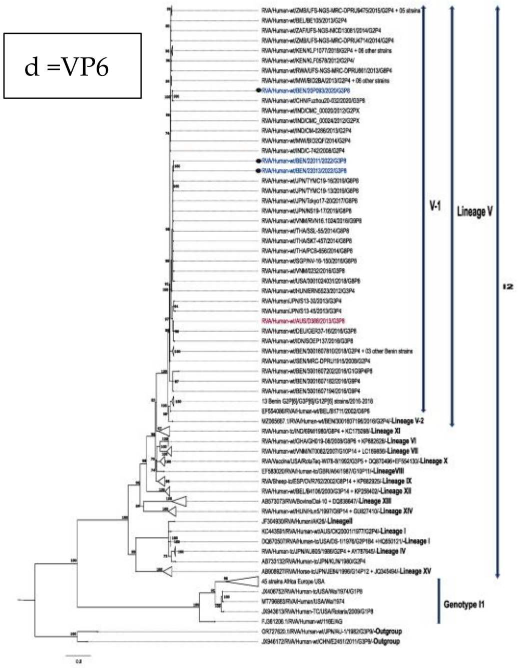

Figure S1: Maximum likelihood phylogenetic tree depicting the genetic relationships of the study strains with other globally circulating strains retrieved from GenBank. The phylogenetic inference for (a) the VP1 gene was performed using the TIM1+G4 evolutionary model ; (b) the VP2 gene was performed using the GTR+G4 evolutionary model ; (c) the VP3 gene was performed using the GTR+I+G4 evolutionary model ; (d) the VP6 gene was performed using the TVM+G4 evolutionary model. Study strains are represented by black circles and are color-coded in blue. The human-derived D388 strain, the prototype of equine-like G3P[8] strains, is color-coded in red. Only bootstrap values of ≥70% are shown at each branch node. The scale bar represents nucleotide substitutions per site.

3.2.4. Phylogenetic analysis of NSP1-5

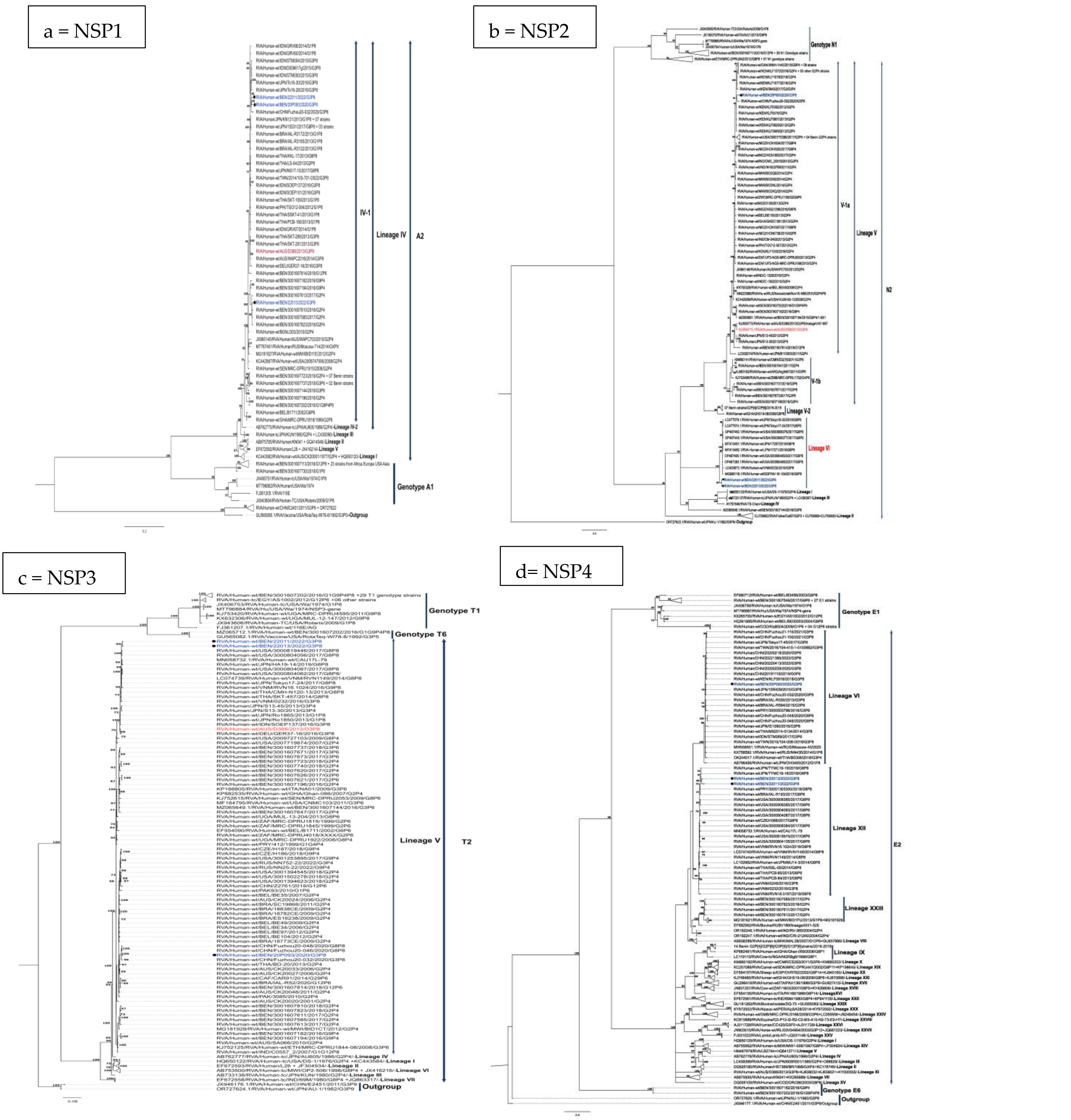
a = NSP1
IV-1
Lineage IV
A2
Genotype A1
b = NSP2
Genotype N1
V-1a
Lineage V
N2
V-1b
Lineage V-2
Lineage VI
c = NSP3
Genotype T1
Genotype T6
Lineage V
T2
Outgroup
d= NSP4
Genotype E1
Lineage VI
Lineage XII
Lineage XXIII
E2
Lineage IX
Genotype E6
Outgroup

e = NSP5

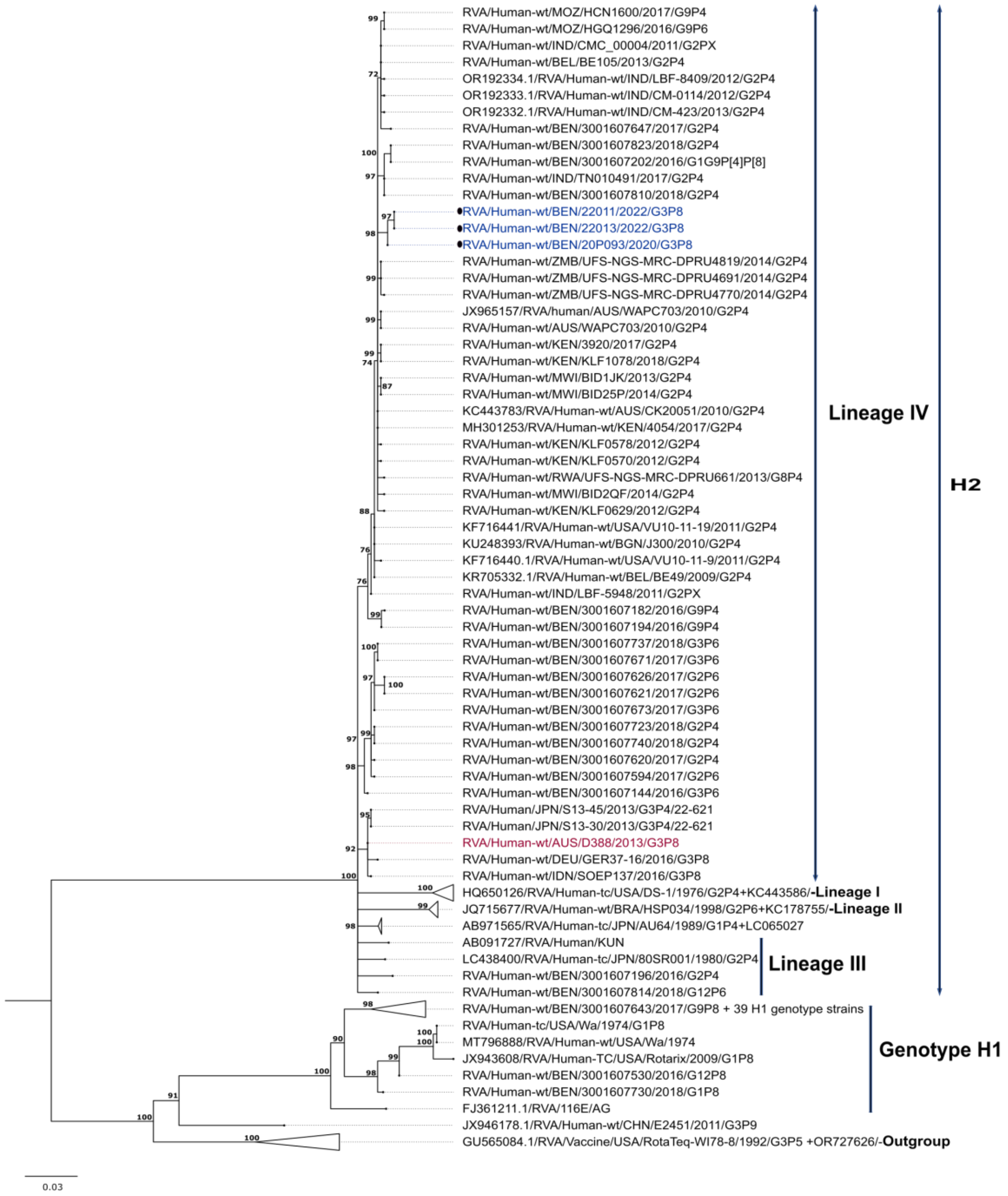

Figure S2: Maximum likelihood phylogenetic tree depicting the genetic relationships of the study strains with other globally circulating strains retrieved from GenBank. The phylogenetic inference for (a) the NSP1 gene was performed using the GTR+I+G4 evolutionary model ; (b) the NSP2 gene was performed using the GTR+G4 evolutionary model ; (c) the NSP3 gene was performed using the TIM1+I+G4 evolutionary model ; (d) the NSP4 gene was performed using the TrN+G4 evolutionary model ; (e) the NSP5 gene was performed using the TPM2uf+G4 evolutionary model. Study strains are represented by black circles and are color-coded in blue. The human-derived D388 strain, the prototype of equine-like G3P[8] strains, is color-coded in red. Only bootstrap values of ≥70% are shown at each branch node. The scale bar represents nucleotide substitutions per site.

Table S1. Metrics on the study strains.

	**RVA/Human-wt/BEN/20P093/2020/G3P8**	**RVA/Human-wt/BEN/22011/2022/G3P8**	**RVA/Human-wt/BEN/22013/2022/G3P8**
Data on raws samples			
Original Reads	550 676	453 602	591 256
Number reads trimming	548 986	452 264	589 316
Read depth using BBNorm	22 204	23 378	29 102
Metrics on contigs following de novo assembly			
Total length of good contigs	14 714	23 665	24 134
Minimal sequence length	300	300	300
Average depth	176.6	132.7	215.6
Average short read coverage	176.6	136.1	145.2
Maximal sequence length	1 714	5 043	5 045
N50	2 286	2 614	2 559
GC%	31.9	32.5	33.7
QC-passed reads after assembly and mapping	14 714	18 488	22 723
QC-failed reads	0	0	0
